# Socioeconomic disparities and regional environment are associated with cervical lymph node metastases in children and adolescents with differentiated thyroid cancer: developing a web-based predictive model

**DOI:** 10.3389/fendo.2024.1128711

**Published:** 2024-02-14

**Authors:** Yaqian Mao, Jinwen Wang, Yinghua Luo, Wei Lin, Jin Yao, Junping Wen, Gang Chen

**Affiliations:** ^1^ Department of Endocrinology, Fujian Provincial Hospital, Shengli Clinical Medical College of Fujian Medical University, Fuzhou, China; ^2^ Fujian Provincial Key Laboratory of Medical Analysis, Fujian Academy of Medical, Fujian, Fuzhou, China

**Keywords:** cervical lymph node metastasis, children and adolescents with differentiated thyroid cancer, regional environment, socioeconomic disparities, web-based predictive model

## Abstract

**Purpose:**

To establish an online predictive model for the prediction of cervical lymph node metastasis (CLNM) in children and adolescents with differentiated thyroid cancer (caDTC). And analyze the impact between socioeconomic disparities, regional environment and CLNM.

**Methods:**

We retrospectively analyzed clinicopathological and sociodemographic data of caDTC from the Surveillance, Epidemiology, and End Results (SEER) database from 2000 to 2019. Risk factors for CLNM in caDTC were analyzed using univariate and multivariate logistic regression (LR). And use the extreme gradient boosting (XGBoost) algorithm and other commonly used ML algorithms to build CLNM prediction models. Model performance assessment and visualization were performed using the area under the receiver operating characteristic (AUROC) curve and SHapley Additive exPlanations (SHAP).

**Results:**

In addition to common risk factors, our study found that median household income and living regional were strongly associated with CLNM. Whether in the training set or the validation set, among the ML models constructed based on these variables, the XGBoost model has the best predictive performance. After 10-fold cross-validation, the prediction performance of the model can reach the best, and its best AUROC value is 0.766 (95%CI: 0.745-0.786) in the training set, 0.736 (95%CI: 0.670-0.802) in the validation set, and 0.733 (95%CI: 0.683-0.783) in the test set. Based on this XGBoost model combined with SHAP method, we constructed a web-base predictive system.

**Conclusion:**

The online prediction model based on the XGBoost algorithm can dynamically estimate the risk probability of CLNM in caDTC, so as to provide patients with personalized treatment advice.

## Background

Thyroid cancer (TC) is the most common malignancy of the endocrine system, and its incidence is increasing worldwide (1, 2). Among them, differentiated thyroid carcinoma (DTC) is the most common subtype of TC, including papillary thyroid carcinoma (PTC) and follicular thyroid carcinoma (FTC), accounting for the vast majority of thyroid malignancies (3). DTC patients are prone to cervical lymph node metastasis (CLNM) and have a higher mortality rate (4). Studies have shown that the presence of cancerous nodules in the lymph node in PTC patients is a new indicator of distant metastasis and poor survival (5). However, considering that lymph node resection can cause laryngeal nerve palsy, hypocalcemia and other surgical complications, it is still controversial whether lymph node dissection should be performed in all patients (6).

Childhood and adolescent DTC (caDTC) is relatively uncommon in the population (7), but its incidence has also been increasing in recent years (8, 9). In a recent study, using data from the Surveillance, Epidemiology, and End Results (SEER) program, reported a gradual increase in the incidence of DTC in children from 1973 to 2006 [annual percentage change (APC), 1.11%; 95% CI, 0.56%-1.67%], of which, increased significantly from 2006 to 2013 (APC, 9.56%; 95%CI, 5.09%-14.22%) (8). Unfortunately, the clinical attention to caDTC is far from enough. The clinical, pathological and molecular features of caDTC differ from adult DTC. Therefore, treatment modalities that work for adults may not necessarily work for children or adolescents (10). Despite the favorable long-term prognosis of caDTC, the risk of recurrence increases significantly once CLNM develops.

TC has increased rapidly over the past 30 years, in addition to being associated with increased rates of diagnosis due to advances in imaging (11–13). Other possible causes are obesity (13)or environmental influences (12). In recent years, ethnicity and socioeconomic status have also been identified as potential reasons for the rapid rise in TC. A recent registry of review results from SEER showed different trends in TC incidence by race/ethnicity, with an increase in TC incidence among those with higher levels of care (2, 14). However, most of these studies focus on adult TC and prognosis, and there is still a lack of reports on the impact of socioeconomic disparities and regional environmental health on caDTC. At present, the research data on the occurrence of CLNM in caDTC is small and not comprehensive enough, and further studies with larger samples are needed for further confirmation.

The current methods for evaluating preoperative lymphatic status mainly include ultrasonography (US), computed tomography (CT) and invasive fine needle aspiration (FNA), but their sensitivity is limited (15, 16). There is currently a lack of more accurate methods to identify the risk of CLNM. Therefore, it is necessary to develop new diagnostic tools to assess the status of cervical lymph nodes. Machine learning (ML) is a new computer-based data analysis method that has been widely used in clinical medicine (17–19). By learning from datasets, ML can discover more interactions between variables and outcomes with better accuracy than traditional statistical methods. Since few studies have established ML prediction models based on caDTC. This study aimed to construct an online computational model for network visualization based on the extreme gradient boosting (XGBoost) algorithm and SHapley Additive exPlanations (SHAP) method to assess the risk of CLNM in caDTC patients. And analyze the impact between socioeconomic disparities, regional environment and CLNM.

## Materials and methods

### Data source and study population

We extracted data from the National Cancer Institute SEER database. The database collects and publishes relevant cancer outcomes for the U.S. population, including demographic characteristics, histological types, TNM stages, and treatment, etc. By registering online, we obtained an access license to the SEER database, access number 11573-Nov2021. We obtained patient information from SEER database through SEER*Stat 8.4.0.1 software (Data source: Incidence-SEER Research Plus Data, 17 Registries, Nov 2021 Sub (2000–2019)), and generated a list of patient information for analysis.

All study subjects met the following inclusion and exclusion criteria: ① Patients with a definite diagnosis of DTC. ② Children and adolescents aged ≤ 18 years old. The following subjects were excluded: ① Data were missing. ② DTC is not the only tumor (ie, combined with other tumors). Since the data used in this study were all publicly available, formal review by the relevant ethics committee was waived. Tumor histological confirmation was performed according to histological codes and topography code C73 in the International Classification of Diseases for Oncology, Third Edition (ICD-O-3). The coding is based on the nomenclature adopted by the World Health Organization (WHO) International Histological Classification of Tumors (Blue Book). The codes for diagnosing PTC include 8050/3, 8260/3, 8340/3, 8341/3, 8342/3, 8343/3, 8344/3 and 8350/3; the codes for diagnosing FTC include 8330/3, 8331/3, 8332/3 and 8335/3. According to the latest definition and classification criteria of WHO (2017), hurthle cell carcinoma (HCC, 8290/3) is considered to have different biological characteristics from FTC, so in this research, we excluded HCC from the study outside.

### Variable screening and classification

Based on extensive literature reading and expert knowledge, we extract the following features from the SEER database. Patients’ socioeconomic demographic information, including age at diagnosis, sex, race, region where the tumor registry is located, median household income, and living conditions. We divided the age of diagnosis into two categories: ≤10 years and 10~18 years. Gender was divided into male and female. Race was divided into white, black and other. Among them, other including American Indian, Alaska Native, Asian or Pacific Islander. According to the region in which the patient’s tumor was registered, it was divided into Pacific Coast (California, Hawaii and Seattle), Eastern (Connecticut、Georgia、Kentucky、Louisiana and New Jersey), Northern Plains (Iowa), and Southwest (New Mexico and Utah). According to the median household income, the family income of patients was divided into 4 categories, namely<5500$, 5500-6500$, 6500-7500$ and>7500$. This is based on 2019 inflation-adjusted U.S. dollars. According to the size of the population and the level of economic development, living conditions can be divided into the following four categories: living in a metropolitan area with a population of 1 million or more, living in a metropolitan area with a population of about 250,000 to 1 million, live in a metropolitan area with a population of less than 250,000, living in non-metropolitan areas.

Detailed variable definitions and classification criteria can be found in **Supplementary Table 1**.

### Model construction and development

We used univariate and multivariate LR analysis to screen for risk factors for developing CLNM in caDTC patients. LR analysis is currently the gold standard for analyzing binary medical data. It can not only assess the risk factors for the occurrence of disease, but also provide the OR value and 95% confidence interval (CI) of the risk factors. We used the feature variables with *P*<0.05 in multivariate LR as modeling variables for ML. Use the XGBoost algorithm for predictive model building and compare with 7 other commonly used ML algorithms. Machine learning methods can be divided into generative methods and discriminant methods. Among the 8 algorithms selected, Gaussian and Bayesian models are generative methods, and the rest are discriminant methods, which makes the comparison benchmark method more comprehensive and the conclusion more convincing. In addition, through a large number of literature reading, we found that the above eight methods are the current common methods of chronic disease prediction model construction. The XGBoost algorithm belongs to the gradient tree boosting framework, which can evaluate a group of weak learners and aggregate them into a strong learner, which is a popular ML method. Model building uses 10-fold cross-validation, which is currently the technique of choice in computer science (20). All samples in the dataset were randomly divided into 10 subsets of similar size with approximately the same and mutually exclusive outcome events. In each round of training, 9 subsets are selected in turn to form the training set, and the remaining 1 subset form the validation set. Each ML model is trained and validated 10 times, each time using a different training and validation set, and the average of the 10 validation results is accepted as the final result.

We assessed model performance by area under the receiver operating characteristic curve (AUROC), sensitivity, specificity, accuracy, positive predictive value (PPV), negative predictive value (NPV), and F1 score. F1 score is an index used to measure the accuracy of the binary classification model in statistics. It takes into account both the accuracy rate and recall rate of the classification model. If the accuracy rate and recall rate are both high, the model will obtain a higher F1 score. We take the ML algorithm with the largest AUROC value as the best model, and further optimize and visualize the best model. Model visualization is mainly done through the Shapley Additive exPlanations (SHAP). SHAP is a framework theory based on additive feature attribution method, first proposed by Lloyd Shapley in game theory (21). For an ensemble tree model, when doing a classification task, the model outputs a probability value. Therefore, SHAP actually attributes the output value to the shapely value of each feature, in other words, calculates the shapely value of each feature, and then measures the impact of the feature on the final output value. In order to determine the importance of each feature to the prediction model, we construct a summary plot based on the XGBoost model. The summary plot plots the SHAP values of all its features for each sample, which provides a better understanding of the overall pattern and allows the discovery of prediction outliers. Each row represents a feature, and the abscissa is the SHAP value.

Based on the XGBoost algorithm and SHAP method, we built a web-based application for identifying caDTC patients at risk for CLNM.

### Statistical analysis

We divided patients into those without lymph node metastasis (LNM) and those with LNM based on whether or not they had CLNM. However, whether CLNM occurs or not is reported according to postoperative pathological findings.

Categorical variables were expressed as frequencies and percentages, and differences in distribution between the two groups were assessed using the chi-square test. We used univariate and multivariate LR analyses to identify risk factors for CLNM and calculate their ORs and 95% Cis, and two-sided *P*<0.05 indicated that the difference was statistically significant. All Statistical analyses were performed using SPSS software (version 25.0 for windows; SPSS Inc., Chicago, IL, USA), R version 4.2.3 and python version 3.11.4. Acknowledgments:This work is supported by Extreme Smart Analysis platform (https://www.xsmartanalysis.com/).

## Results

### Baseline characteristics

A total of 2519 patients with caDTC aged ≤ 18 years were included in this study. There were 445 male subjects and 2074 female subjects, and a total of 1279 patients developed CLNM. In this cohort, 32.31% of patients had a median household income of more than 7,500$, 24.18% had a median household income of 6,500-7,500$, and 23.82% had a median household income of 5,500-6,500$, 19.69% of patients had a median household income of less than 5,500$. Most of these patients live in metropolitan areas with a population of 1 million (see **Supplementary Table 2** for details).

### Risk factors associated with cervical lymph node metastasis

The results of univariate LR analysis showed that in addition to living conditions, age, multifocality, race, sex, histological type, extrathyroidal extension (ETE), tumor size, region and median household income were closely related to the risk of CLNM in caDTC. We included risk factors with *P*<0.05 in the univariate LR analysis into the multivariate LR analysis (see **Table 1** for details).

**Table 1:** Univariate and multivariate LR analyses of CLNM.

Characteristics	Univariate Analysis		Multivariate Analysis	
	OR, 95%CI	*P* Value	OR, 95%CI	*P* Value
Age (years)				
≤10	1 [Reference]		1 [Reference]	
10-18	0.465 (0.332-0.651)	<0.001*	0.441 (0.297-0.654)	<0.001*
Race				
White	1 [Reference]		1 [Reference]	
Black	0.566 (0.379-0.845)	0.005*	0.617 (0.388-0.982)	0.042*
Other^a^	1.011 (0.788-1.298)	0.931	0.796 (0.597-1.060)	0.118
Sex				
Female	1 [Reference]		1 [Reference]	
Male	1.346 (1.095-1.655)	0.005*	1.224 (0.968-1.549)	0.092
Histological type				
PTC	1 [Reference]		1 [Reference]	
FTC	0.017 (0.006-0.046)	<0.001*	0.015 (0.006-0.042)	<0.001*
ETE				
Intrathyroidal extension/mETE^b^	1 [Reference]		1 [Reference]	
gETE	9.993 (5.725-17.441)	<0.001*	6.477 (3.571-11.747)	<0.001*
Tumor size (cm)				
≤1	1 [Reference]		1 [Reference]	
1-2	2.186 (1.711-2.793)	<0.001*	2.451 (1.894-3.172)	<0.001*
2-4	2.414 (1.906-3.057)	<0.001*	2.822 (2.190-3.637)	<0.001*
>4	3.389 (2.572-4.464)	<0.001*	4.152 (3.032-5.685)	<0.001*
Mulifocality				
Solitary tumor	1 [Reference]		1 [Reference]	
Multifocal tumor	3.052 (2.505-3.719)	<0.001*	2.477 (2.005-3.060)	<0.001*
Unknown	1.760 (1.452-2.132)	<0.001*	1.450 (1.167-1.802)	0.001*
Region^c^				
Pacific Coast	1 [Reference]		1 [Reference]	
East	0.611 (0.516-0.724)	<0.001*	0.662 (0.543-0.805)	<0.001*
Northern Plains	0.705 (0.474-1.049)	0.085	0.828 (0.530-1.293)	0.406
Southwest	0.978 (0.719-1.331)	0.888	1.063 (0.749-1.508)	0.734
Median household income				
<55,000$	1 [Reference]		1 [Reference]	
55,000-64,999$	1.351 (1.064-1.715)	0.013*	1.133 (0.861-1.491)	0.372
65,000-74,999$	1.202 (0.948-1.525)	0.129	1.105 (0.844-1.449)	0.467
≥75,000$	1.401 (1.119-1.753)	0.003*	1.331 (1.025-1.728)	0.032*
Living conditions				
Metropolitan areas (1 million or more)	1 [Reference]			
Metropolitan areas (250,000 to 1 million)	0.915 (0.753-1.112)	0.372		
Metropolitan areas (less than 250,000)	0.780 (0.585-1.041)	0.092		
Nonmetropolitan counties^d^	0.933 (0.712-1.222)	0.612		

a Other including American Indian, Alaska Native, Asian or Pacific Islander;

b Intrathyroidal extension/mETE including limited to the thyroid, or any tumor with minimal extrathyroid extension;

c Region: Pacific coast including California, Hawaii and Seattle; East including Connecticut、Georgia、Kentucky、Louisiana and New Jersey; Northern plains including Iowa; Southwest including New Mexico and Utah;

d Nonmetropolitan counties including nonmetropolitan adjacent to a metropolitan area and nonmetropolitan counties not adjacent to a metropolitan area.

CLNM, Cervical lymph node metastasis; PTC, Papillary thyroid carcinoma; FTC, Follicular thyroid carcinoma; ETE, Extrathyroid extension; mETE, Minimal extrathyroidal extension; gETE, Gross extrathyroidal extension.

The symbol * indicates *P* < 0.05.

In the multivariate LR analysis (**Table 1**), there were significant statistical differences in all variables except sex (all *P*<0.05). The OR value of the adolescent group (10-18years) was lower than that of the children group (≤10years; OR=0.441, 95%CI: 0.297-0.654, *P*<0.001). The FTC group had a lower OR value compared with that of the PTC group (OR=0.015, 95%CI: 0.006-0.042, *P*<0.001). Patients residing in the Eastern United States had a lower risk of developing cervical LNM than those residing in the Pacific Coast (OR=0.662, 95%CI: 0.543-0.805, *P*<0.001). Multifocality, larger tumors, and more extracapsular invasion of the thyroid are associated with a higher risk of developing CLNM. Among racial types, blacks had a reduced risk of developing CLNM compared with whites.

More importantly, we found that people with higher median household income (≥75,000$) had a higher risk of developing CLNM than those with lower median household income (35,000-54,999$) (OR=1.331, 95%CI: 1.025-1.728, *P*=0.032*). To further understand the reasons for this distributional difference, we analyzed the impact of region, ethnicity, and living conditions on caDTC household income (see **Table 2** for details). We can see that patients with a median household income>7,500$ mainly live in metropolitan areas with a population of about 1 million. Among them, 98.16% of the population lives in the economically developed areas of the Pacific Coast and East. The proportions of minority (American Indian, Alaska Native, Asian or Pacific Islander) are relatively higher in these regions.

**Table 2:** Effect of region、race and living conditions on median household income in caDTC.

Category	Median household income				*X* ^2^	*P*
	<5500$	5500-6500$	6500-7500$	>7500$		
Living conditions						
Metropolitan areas (1 million or more)	45(9.073)	376(62.667)	429(70.443)	663(81.450)	1051.926	<0.001*
Metropolitan areas (250,000 to 1 million)	148(29.839)	113(18.833)	150(24.631)	138(16.953)		
Metropolitan areas (less than 250,000)	116(23.387)	76(12.667)	17(2.791)	3(0.369)		
Nonmetropolitan counties	187(37.702)	35(5.833)	13(2.135)	10(1.229)		
Region^a^						
Pacific Coast	127(25.605)	367(61.167)	316(51.888)	452(55.528)	290.07	<0.001*
East	273(55.040)	147(24.500)	196(32.184)	347(42.629)		
Northern Plains	37(7.460)	47(7.833)	21(3.448)	1(0.123)		
Southwest	59(11.895)	39(6.500)	76(12.479)	14(1.720)		
Race						
White	450(90.726)	507(84.500)	533(87.521)	643(78.993)	89.209	<0.001*
Black	30(6.048)	34(5.667)	22(3.612)	21(2.580)		
Other^b^	16(3.226)	59(9.833)	54(8.867)	150(18.428)		

a Region: Pacific coast including California, Hawaii and Seattle; East including Connecticut、Georgia、Kentucky、Louisiana and New Jersey; Northern plains including Iowa; Southwest including New Mexico and Utah.

b Other including Asian or Pacific Islander, American Indian/Alaska Native.

The symbol * indicates *P* < 0.05.

### Machine learning model construction and screening

**Figure 1:**
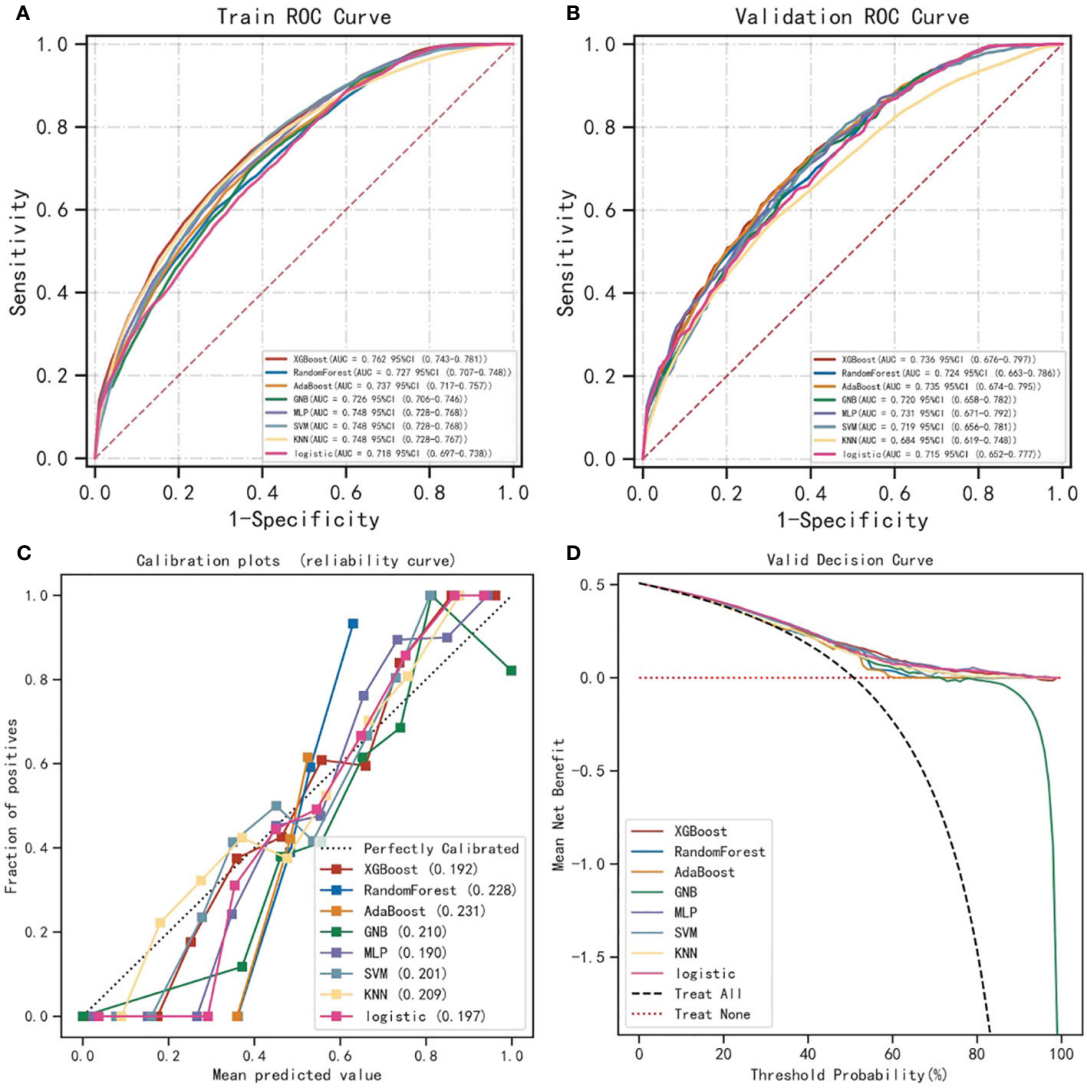
Performance comparison of XGBoost algorithm and other ML algorithms in predicting lymph node metastasis. **(A, B)** compare the performance of 8 different ML algorithms in building predictive models. Whether in the training set or the validation set, the XGBoost algorithm has the highest AUROC value and is the best predictive model. **(C)** is the calibration curve of the prediction model. The abscissa of the graph is the predicted probability, that is, the probability of the event occurrence is predicted by the prediction model. The ordinate is the actual probability, that is, the patient’s actual event rate. Each colored solid line is a fitted line, representing the actual value corresponding to the predicted value. If the predicted value is equal to the actual value, the solid line exactly coincides with the diagonal dashed line. **(D)** shows the decision curve analysis of each model. The results of the study showed that the population using the ML model benefited well. ML, Machine learning; XGBoost, Extreme gradient boosting; AUROC, Area under the receiver operating characteristic.

We used variables with *P*<0.05 in multivariate LR analysis for 8 different ML model constructions. The predictive model was constructed using a 10-fold cross-validation method. The parameter settings of each model are shown in **Supplementary Table 3**. Whether in the training set or the validation set, the XGBoost algorithm has the highest AUROC value and is the best predictive model (see **Table 3** for details, **Figures 1A, B**). Its AUROC value is 0.762 (95%CI: 0.743-0.781) in the training set and 0.736 (95%CI: 0.676-0.797) in the validation set. **Figure 1C** shows calibration curves for different ML models. It can be seen from the figure that the calibration curves of these ML models are in good agreement with the reference line, that is, the diagonal line, indicating that the predicted values estimated by these models are in the best agreement with the actual values. **Figure 1D** shows the decision curve analysis of each model, and the results show that the population estimated using these models has a good benefit.

**Table 3:** Comparison of prediction performance of 8 different machine learning models.

ML	AUROC (95%CI)	Accuracy (95%CI)	Sensitivity (95%CI)	Specificity (95%CI)	PPV (95%CI)	NPV (95%CI)	F1-score (95%CI)
Training set							
XGBoost*	0.762 (0.743-0.781)	0.686 (0.683-0.688)	0.695 (0.661-0.729)	0.678 (0.645-0.712)	0.692 (0.680-0.705)	0.683 (0.670-0.696)	0.692 (0.681-0.703)
**SVM**	0.748 (0.728-0.768)	0.683 (0.681-0.686)	0.759 (0.749-0.769)	0.608 (0.597-0.619)	0.666 (0.663-0.670)	0.707 (0.702-0.713)	0.710 (0.706-0.713)
**RF**	0.727 (0.707-0.748)	0.659 (0.656-0.662)	0.657 (0.617-0.698)	0.663 (0.626-0.700)	0.673 (0.663-0.682)	0.649 (0.637-0.661)	0.663 (0.646-0.679)
**MLP**	0.748 (0.728-0.768)	0.674 (0.669-0.678)	0.654 (0.627-0.681)	0.697 (0.676-0.717)	0.690 (0.683-0.696)	0.661 (0.650-0.672)	0.670 (0.658-0.682)
**LR**	0.718 (0.697-0.738)	0.649 (0.647-0.651)	0.741 (0.696-0.785)	0.557 (0.512-0.601)	0.635 (0.624-0.645)	0.677 (0.659-0.695)	0.681 (0.667-0.695)
**KNN**	0.748 (0.728-0.767)	0.669 (0.666-0.672)	0.653 (0.646-0.660)	0.710 (0.701-0.718)	0.731 (0.724-0.738)	0.631 (0.626-0.636)	0.690 (0.686-0.693)
**GNB**	0.726 (0.706-0.746)	0.663 (0.660-0.666)	0.706 (0.691-0.721)	0.623 (0.609-0.636)	0.659 (0.655-0.663)	0.669 (0.662-0.676)	0.682 (0.676-0.687)
**AdaBoost**	0.737 (0.717-0.757)	0.665 (0.663-0.666)	0.665 (0.642-0.688)	0.670 (0.649-0.691)	0.679 (0.677-0.682)	0.651 (0.647-0.655)	0.672 (0.661-0.682)
Validation set							
XGBoost*	0.736 (0.676-0.797)	0.667 (0.652-0.682)	0.709 (0.641-0.777)	0.660 (0.588-0.731)	0.674 (0.654-0.694)	0.663 (0.648-0.679)	0.686 (0.655-0.717)
**SVM**	0.719 (0.656-0.781)	0.660 (0.643-0.677)	0.738 (0.665-0.811)	0.623 (0.550-0.695)	0.646 (0.631-0.661)	0.682 (0.657-0.706)	0.685 (0.648-0.721)
**RF**	0.724 (0.663-0.786)	0.651 (0.636-0.666)	0.678 (0.586-0.769)	0.658 (0.572-0.744)	0.666 (0.644-0.687)	0.641 (0.624-0.659)	0.664 (0.623-0.706)
**MLP**	0.731 (0.671-0.792)	0.653 (0.629-0.678)	0.708 (0.629-0.788)	0.655 (0.566-0.744)	0.665 (0.643-0.687)	0.643 (0.616-0.670)	0.680 (0.641-0.720)
**LR**	0.715 (0.652-0.777)	0.630 (0.606-0.655)	0.769 (0.690-0.849)	0.565 (0.473-0.656)	0.616 (0.596-0.637)	0.658 (0.621-0.695)	0.680 (0.644-0.717)
**KNN**	0.684 (0.619-0.748)	0.626 (0.602-0.651)	0.675 (0.572-0.779)	0.610 (0.492-0.729)	0.678 (0.642-0.714)	0.596 (0.577-0.615)	0.664 (0.611-0.716)
**GNB**	0.720 (0.658-0.782)	0.661 (0.638-0.684)	0.777 (0.718-0.835)	0.576 (0.501-0.651)	0.656 (0.637-0.675)	0.667 (0.639-0.696)	0.708 (0.685-0.731)
**AdaBoost**	0.735 (0.674-0.795)	0.663 (0.650-0.675)	0.650 (0.588-0.712)	0.707 (0.655-0.759)	0.678 (0.663-0.692)	0.649 (0.637-0.661)	0.660 (0.624-0.697)

*indicated that the best performance of the ML classifier in the training set and validation sets was XGBoost (Ranked according to AUC).

ML, Machine learning; XGBoost, Extreme gradient boosting; RF, Random forest; AdaBoost, Adaptive boosting; GNB, Gaussian naive bayes; MLP, Multilayer perceptron; SVM, Support vector machine; KNN, k-nearest neighbor; LR, Logistic regression; AUROC, Area under the receiver operating characteristic curve; PPV, positive predictive value; NPV, Negative predictive value; CI, Confidence interval.

### Web-based application system development

**Figure 2:**
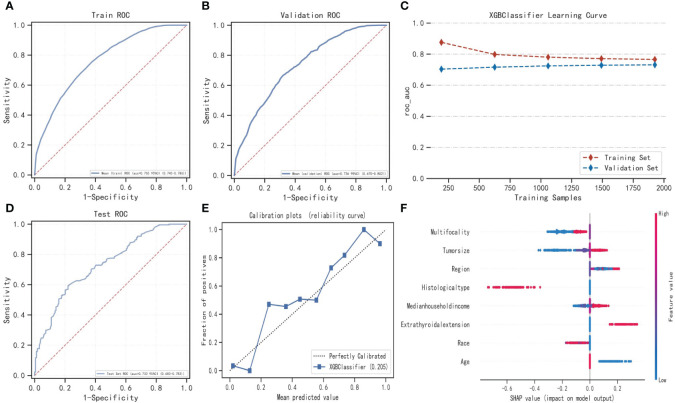
Construction of LNM prediction model based on XGBoost algorithm and SHAP method. **(A–D)** show the optimization process of the XGBoost model based on 10-fold cross-validation. When the learning curves of the training set and validation set converge **(C)**, the prediction performance of the XGBoost model is the best at this time. **(E)** is the calibration curve based on the XGBoost model. **(F)** shows SHAP based on the XGBoost model. XGBoost, Extreme gradient boosting; ROC, Receiver operating characteristic; SHAP, Shapley Additive exPlanations.

We used the XGBoost algorithm with the best predictive performance for the visualization of the predictive model and the development of the web application system (**Figure 2**). We randomly selected 15% of the data in the total sample as the test set (N=377). The remaining samples were used as training set and validation set for 10-fold cross-validation. The model has AUC=0.766 (95%CI: 0.745-0.786) in the training set, AUC=0.736 (95%CI: 0.670-0.802) in the validation set, and AUC=0.733 (95%CI: 0.683-0.783) in the test set. **Figure 2F** shows SHAP based on XGBoost algorithm.

**Figure 3:**
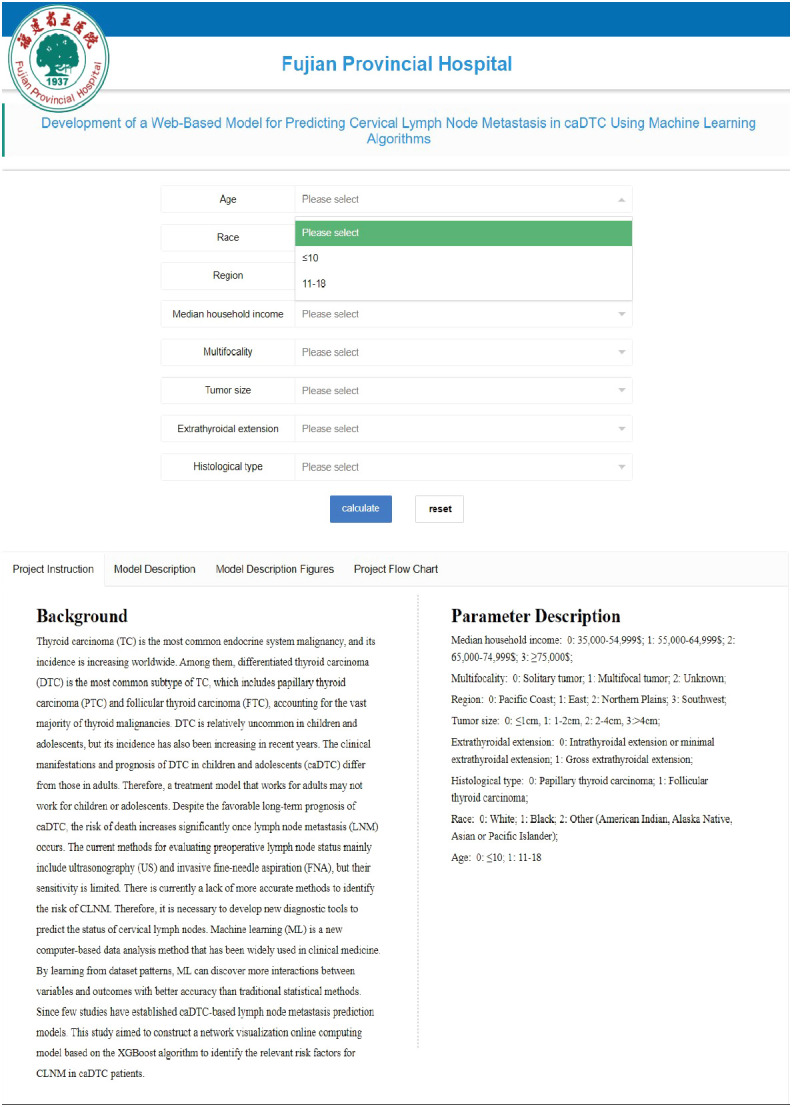
Web-based visual risk prediction model page for CLNM in caDTC.

This application has a friendly interface (**Figure 3**). The user only needs to enter 8 variables in the web browser, and the specific values of these variables are selected from the drop-down list. Once the doctor submits the data, the app provides probabilistic information about the risk of LNM and provides advice. The web link of the web application system is https://www.xsmartanalysis.com/model/predict/?mid=1171&symbol=3rOeoUn1660006924zB6.

## Discussion

In response to the current elevated incidence of TC, adding reliable and easy-to-use CLNM prediction models will allow clinicians and intelligent systems to better make evidence-based patient care decisions. This study has developed and internally validated a web-based model to predict the risk of developing CLNM in patients with caDTC. The study population was obtained from patients registered with multiple cancer centers in the US SEER database. In this study, we constructed an online computational model of risk for the caDTC network visualization, using the XGBoost algorithm and the SHAP method, based on a large cohort (2,519 cases). The model consisted of eight main risk factors, including age, race, histological type, tumor size, ETE, multiple foci, area of residence, and median household income. Compared with other large studies, in addition to having similar CLNM-related risk factors in this study, we found that socioeconomic factors and regional environment had a significant effect on the performance of caDTC patients. To the best of our knowledge, this is a larger comprehensive study and intelligent model construction on caDTC patients and socioeconomic differences.

A growing number of studies have shown that socioeconomic status has a critical impact on TC prognosis. A study from Almubarak et al (22)found that living in a rural area (*P*<0.001) and low literacy (*P*=0.021) were significantly associated with the onset of late stage TC. This study suggests that even in a country like Saudi Arabia, which has a strong government-funded healthcare system, there are health disparities among people struggling with TC, with patients in the low socioeconomic status group often being diagnosed at a more advanced stage at the time of presentation. Swegal et al (23)also showed that in addition to the effect on incidence, low socioeconomic status was assciated with poorer survival in highly differentiated thyroid cancer (WDTC). A study by Harari et al (24)also confirmed that TC patients of black and low socioeconomic status had worse outcomes. The effect of socioeconomic status on the incidence and prognosis of WDTC has been well studied. However, the relationship between socioeconomic level and CLNM has not been described. Our findings seem to lead to the opposite conclusion that patients with caDTC with higher household income (≥75,000$) are more likely to develop CLNM. to further understand the reasons for this distributional difference, we analyzed the effects of region, race and living conditions on household income in caDTC. We could see that patients with median household income >75,000$ lived mainly in metropolitan areas with a population of about 1 million. Of this group, 98.16% live in economically developed areas such as the Pacific Coast and East. The percentage of minorities (American Indian, Alaska Native, Asian or Pacific Islander) was relatively higher in these areas. The reasons for these factors may be related to the following factors: ① Higher socioeconomic status is more likely to have access to good medical resources and relatively higher levels of CLNM detection. ② Our study also found that patients with higher average income levels tend to live closer to large cities. These places, in turn, may have more environmental pollution compared to remote rural areas, and TC is a class of diseases related to environmental health. This may also explain to some extent why the prevalence of TC is much higher in areas with developed economic levels compared to less developed areas. ③ In recent years, it has also been shown that obesity and high body mass index (BMI) are strongly associated with the occurrence of TC (25, 26), and obesity is also more prevalent in areas with high economic income. This may, to some extent, explain the greater risk of CLNM among those with median income>75,000$ in this study. The multifactorial LR analysis in this study showed a lower risk of CLNM in blacks compared to whites, which may also be related to the higher economic level and BMI possessed by whites. This is one of the limitations of this study as we were unable to assess the BMI of the patients in this study.

With the continuous development of science and technology, ML has brought great convenience to our life. However, there is limited research using ML algorithms to predict the occurrence of cervical LNM in TC, especially in the application to patients with caDTC. ML uses algorithms to process and reveal patterns in large amounts of data to develop predictive models that automatically improve over time. A growing number of studies are predicting the risk of disease by constructing Web-based models. Such Web-based health care content has become a primary source of health information for patients without direct guidance from health care providers (27).

In this study, we combined the XGBoost algorithm and SHAP method to construct an online computational model of the network for the occurrence of CLNM in caDTC. By integrating eight risk factors, we can estimate the risk of developing CLNM in a particular caDTC patient and provide treatment advice. By entering personalized information, patients can share this information with family members, and this approach can reduce the anxiety level of patients’ families to some extent. It is well known that different patients require different healthcare content and that it changes over time. In addition, another advantage of personalized healthcare content is its potential to improve health-related choices, i.e., shared responsibility for clinical decisions with patients (shared decision making) (28). Such Web-based personalized predictive models and decision support have been shown to improve patient knowledge and facilitate decisions that are more aligned with patient values and preferences (29, 30).

This study has the following advantages: ① This is the first study of ML algorithm-based models to predict the occurrence of CLNM in patients with caDTC. It shows that these ML-based models have good predictive performance and clinical application, among which the XGBoost model has the best predictive performance. ② We constructed an online application model for the risk of developing CLNM in patients with caDTC based on the XGBoost algorithm and the SHAP method, which compensates for the unexplainable limitations of ML black-box operation. ③ There are few studies on caDTC. In addition to the known risk factors, we found that socioeconomic factors and regional environment have a significant impact on the performance of patients with caDTC. In addition there are some limitations of this study: ① This study is a retrospective cohort study and data bias cannot be avoided. It needs to be validated in future studies through rigorously designed prospective studies. ② In a study of this size, false positive results may occur by statistical chance alone. However, our shorter confidence intervals imply a higher precision and accuracy in estimating the effect. ③ Our regression analysis of risk factors that may explain CLNM also has some limitations, such as obesity, family history, exposure to radiation/carcinogens, and lifestyle. Unfortunately, we did not obtain this information from the registry, so we were unable to assess whether these factors had an impact on the adjusted analysis.

## Conclusions

An online prediction model constructed based on the XGBoost algorithm can dynamically estimate the risk probability of developing CLNM in caDTC thus providing personalized treatment advice for patients. Socioeconomic disparities and regional environment have a significant impact on the performance and outcome of caDTC.

## Data availability statement

The raw data supporting the conclusions of this article will be made available by the authors, without undue reservation.

## Ethics statement

Since the data used in this study were all publicly available, formal review by the relevant ethics committee was waived. The studies were conducted in accordance with the local legislation and institutional requirements.

## Author contributions

GC has full access to all of the data in the study and takes responsibility for the integrity of the data and the accuracy of the data analysis. Concept and design: YM and GC. Acquisition, analysis, or interpretation of data: JWW, YL, and YM. Drafting of the manuscript: YM, WL, JY, JPW, and GC. Critical revision of the manuscript for important intellectual content: YM, JWW, and YL. Statistical analysis: YM, JWW, and YL. Supervision: GC. All authors contributed to the article and approved the submitted version.
