# Supplementary material for: Socioeconomic disparities and regional environment are associated with cervical lymph node metastases in children and adolescents with differentiated thyroid cancer: developing a web-based predictive model
Source: Front Endocrinol (Lausanne). 2024 Feb 14;15:1128711. doi: 10.3389/fendo.2024.1128711 (PMC10916284; doi:10.3389/fendo.2024.1128711)
Supplement: Supplementary file 1 [file Table_1.docx]

**Table S1. Definition, classification and assignment of characteristic variables**

| **Variables** | **Classification** | **Assignment** |
| --- | --- | --- |
| **Cervical lymph node metastasis** | 0~1 | 0: No; 1: Yes |
| **Age (years)** | 0~1 | 0:≤10; 1: 10-18 |
| **Sex** | 0~1 | 0: Female; 1: Male |
| **Race** | 0~2 | 0: White; 1: Black; 2: Other (American Indian, Alaska Native, Asian or Pacific Islander) |
| **Histological type** | 0~1 | 0: PTC: 8050/3: Papillary carcinoma, NOS; 8260/3: Papillary adenocarcinoma, NOS; 8340/3: Papillary carcinoma, follicular variant; 8341/3: Papillary microcarcinoma; 8342/3: Papillary carcinoma, oxyphilic cell; 8343/3: Papillary carcinoma, encapsulated; 8344/3: Papillary carcinoma, columnar cell; 8350/3: Nonencapsulated sclerosing carcinoma;  1: FT**C**: 8330/3: Follicular adenocarcinoma, NOS; 8331/3: Follicular adenocarcinoma well differentiated; 8332/3: Follicular adenocarcinoma trabecular; 8335/3: Follicular carcinoma, minimally invasive |
| **ETE** | 0~1 | 0: Intrathyroidal extension or minimal extrathyroidal extension (mETE) ^a^;  1: Gross extrathyroidal extension (gETE) |
| **Tumor size (cm)** | 0~3 | 0: ≤1cm, 1: 1-2cm, 2: 2-4cm, 3: ＞4cm |
| **Region** | 0~3 | 0: Pacific Coast (California, Hawaii and Seattle);  1: East (Connecticut、Georgia、Kentucky、Louisiana and New Jersey);  2: Northern Plains (Iowa);  3: Southwest (New Mexico and Utah) |
| **Multifocality** | 0~2 | 0: Solitary tumor; 1: Multifocal tumor; 2: Unknown |
| **Median household income** | 0~3 | 0: <55,000$, 1: 55,000-64,999$, 2: 65,000-74,999$, 3: ≥75,000$ |
| **Living conditions** | 0~3 | 0: Metropolitan areas (1 million or more);  1: Metropolitan areas (250,000 to 1 million);  2: Metropolitan areas (less than 250,000);  3: Nonmetropolitan counties^b^ |

**Note**: ^a^Intrathyroidal extension/mETE including limited to the thyroid, or any tumor with minimal extrathyroid extension; ^b^Nonmetropolitan counties including nonmetropolitan adjacent to a metropolitan area and nonmetropolitan counties not adjacent to a metropolitan area.

Since 1988, SEER has recorded extrathyroidal extension (ETE) using 3 different protocols: Extent of Disease 10-extent (EOD 10-extent) codes for 1988-2003, Collaborative Staging extension (CS extension) codes for 2004-2015, and Extent of Disease Primary Tumor (EOD Primary Tumor) codes for 2018+. Combine these codes with Derived SEER Combined T codes for 2016-2017 for ETE classification of TC cases diagnosed in 2000-2019. Depending on whether TC has extracapsular extension, we classify it into the following two categories: Tumor confined to the thyroid or minimal extrathyroidal extension (mETE) and gross extrathyroidal extension (gETE). mETE refers to tumors infiltrating only very small extrathyroidal strap muscle, such as the scapulohyoid, sternohyoid, sternothyroid, or thyrohyoid muscles. gETE refers to the tumor invading the strap muscle, but also invading the peripheral thyroid nerves, blood vessels, tissues and organs, such as recurrent laryngeal nerve, vagus nerve, carotid artery, jugular vein, thyroid artery and vein, esophagus, sternocleidomastoid muscle, etc. Described as being immobilized on adjacent tissue.

Since 1988, SEER has recorded tumor size using 3 different protocols: Extent of Disease 10-size (EOD 10-size) codes for 1988-2003, Collaborative Staging (CS tumor size) codes for 2004-2015, and Tumor Size Summary codes for 2016+. Combined with these codes, TC cases diagnosed in 2000-2019 were size-classified according to tumor size. We classified tumor size into <1cm, 1-2cm, 2-4cm and≥4cm. We classified whether TC was multifocal or not according to Collaborative Staging site-specific factor 1 codes for 2004-2017. According to whether the tumor is multifocal, it is divided into solitary tumor and multifocal tumor. Due to incomplete information on tumor multifocality in some years, we recorded missing data as unknown. According to whether there is lymph node metastasis, it is divided into no and yes.
