# Supplementary material for: Socioeconomic disparities and regional environment are associated with cervical lymph node metastases in children and adolescents with differentiated thyroid cancer: developing a web-based predictive model
Source: Front Endocrinol (Lausanne). 2024 Feb 14;15:1128711. doi: 10.3389/fendo.2024.1128711 (PMC10916284; doi:10.3389/fendo.2024.1128711)
Supplement: Supplementary file 2 [file Table_2.docx]

**Table S2. Baseline characteristics of children and adolescents with differentiated thyroid cancer, n (%)**

| **Characteristics** | **Total**  (N=2519) | **Without CLNM**  (N=1240) | **With CLNM**  (N=1279) | **X^2^** | ***P*** |
| --- | --- | --- | --- | --- | --- |
| **Age (years)** |  |  |  | 20.668 | <0.001 |
| ≤10 | 165(6.550) | 53(4.274) | 112(8.757) |  |  |
| 10-18 | 2354(93.450) | 1187(95.726) | 1167(91.243) |  |  |
| **Gender** |  |  |  | 7.993 | 0.005 |
| Female | 2074(82.334) | 1048(84.516) | 1026(80.219) |  |  |
| Male | 445(17.666) | 192(15.484) | 253(19.781) |  |  |
| **Race** |  |  |  | 8.025 | 0.018 |
| White | 2133(84.676) | 1038(83.710) | 1095(85.614) |  |  |
| Black | 107(4.248) | 67(5.403) | 40(3.127) |  |  |
| Other^a^ | 279(11.076) | 135(10.887) | 144(11.259) |  |  |
| **Histological type** |  |  |  | 201.953 | <0.001 |
| PTC | 2323(92.219) | 1048(84.516) | 1275(99.687) |  |  |
| FTC | 196(7.781) | 192(15.484) | 4(0.313) |  |  |
| **Tumor size (cm)** |  |  |  | 85.674 | <0.001 |
| ≤1 | 451(17.904) | 304(24.516) | 147(11.493) |  |  |
| 1-2 | 722(28.662) | 351(28.306) | 371(29.007) |  |  |
| 2-4 | 908(36.046) | 419(33.790) | 489(38.233) |  |  |
| ＞4 | 438(17.388) | 166(13.387) | 272(21.267) |  |  |
| **ETE** |  |  |  | 96.389 | <0.001 |
| Intrathyroidal extension/mETE**^b^** | 2374(94.244) | 1226(98.871) | 1148(89.758) |  |  |
| gETE | 145(5.756) | 14(1.129) | 131(10.242) |  |  |
| **Multifocality** |  |  |  | 129.614 | <0.001 |
| Solitary tumor | 1164(46.209) | 702(56.613) | 462(36.122) |  |  |
| Multifocal tumor | 686(27.233) | 228(18.387) | 458(35.809) |  |  |
| Unknown | 669(26.558) | 310(25.000) | 359(28.069) |  |  |
| **Region**^c^ |  |  |  | 34.939 | <0.001 |
| Pacific coast | 1262(50.099) | 557(44.919) | 705(55.121) |  |  |
| East | 963(38.229) | 543(43.790) | 420(32.838) |  |  |
| Northern plains | 106(4.208) | 56(4.516) | 50(3.909) |  |  |
| Southwest | 188(7.463) | 84(6.774) | 104(8.131) |  |  |
| **Median household income** |  |  |  | 9.898 | 0.019 |
| <55,000$ | 496(19.690) | 272(21.935) | 224(17.514) |  |  |
| 55,000-64,999$ | 600(23.819) | 284(22.903) | 316(24.707) |  |  |
| 65,000-74,999$ | 609(24.176) | 306(24.677) | 303(23.690) |  |  |
| ≥75,000$ | 814(32.314) | 378(30.484) | 436(34.089) |  |  |
| **Living condition** |  |  |  | 3.246 | 0.355 |
| Metropolitan areas  (about 1 million people) | 1513(60.064) | 727(58.629) | 786(61.454) |  |  |
| Metropolitan areas  (250,000 to 1 million people) | 549(21.794) | 276(22.258) | 273(21.345) |  |  |
| Metropolitan areas  (about 250,000 people) | 212(8.416) | 115(9.274) | 97(7.584) |  |  |
| Nonmetropolitan counties^d^ | 245(9.726) | 122(9.839) | 123(9.617) |  |  |

**Note**: ^a^Other including American Indian, Alaska Native, Asian or Pacific Islander; ^b^Intrathyroidal extension/mETE including limited to the thyroid, or any tumor with minimal extrathyroid extension; ^c^Region: Pacific coast including California, Hawaii and Seattle (Puget Sound); East including Connecticut、Georgia、Kentucky、Louisiana and New Jersey; Northern Plains including Iowa; Southwest including New Mexico and Utah; ^d^Nonmetropolitan counties including nonmetropolitanadjacent to a metropolitan area or nonmetropolitan counties not adjacent to a metropolitan area.

**Abbreviation**: **CLNM**: Cervical lymph node metastasis; **PTC**: Papillary thyroid carcinoma; **FTC**: Follicular thyroid carcinoma; **ETE**: Extrathyroid extension; **mETE**: Minimal extrathyroidal extension; **gETE**: Gross extrathyroidal extension.
