# Supplementary material for: Socioeconomic disparities and regional environment are associated with cervical lymph node metastases in children and adolescents with differentiated thyroid cancer: developing a web-based predictive model
Source: Front Endocrinol (Lausanne). 2024 Feb 14;15:1128711. doi: 10.3389/fendo.2024.1128711 (PMC10916284; doi:10.3389/fendo.2024.1128711)
Supplement: Supplementary file 3 [file Table_3.docx]

**Table S3. Parameter settings and model description for 8 different machine learning algorithms**

| **Model type** | **Parameter settings** | **Model description** |
| --- | --- | --- |
| **XGBoost** | objective: binary:logistic learning_rate: 0.3 max_depth: 2 min_child_weight: 1 reg_lambda: 1 | Extreme gradient boosting (XGBoost) is an improved algorithm based on gradient boosting decision trees (GBDT), which can efficiently construct boosted trees and realize parallel operation. This algorithm can quickly run large-scale data, and is good at dealing with irregular data with many outliers and missing values. |
| **RF** | criterion: gini max_depth: 1 min_impurity_decrease: 0 n_estimators: 50 | Random forests (RF) is an ensemble learning algorithm, which is a combination recognition model formed by combining multiple decision trees. The prediction accuracy of RF classification is relatively high, it is not easy to overfit, and it has strong anti-noise ability and is easy to implement. In addition, RF can also provide information on the importance of categorical variables, so it has received extensive attention. |
| **AdaBoost** | learning_rate: 0.1 n_estimators: 50 | Adaptive boosting (AdaBoost) algorithm is a boost algorithm proposed by Freund et al. Its core idea is to train different classifiers (weak classifiers) for the same training set, and then group these weak classifiers to form a stronger final classifier (strong classifier). |
| **GNB** | priors: None var_smoothing（var_smoothing）: 1e-09 | The gaussian naive bayes (GNB) algorithm is a simple classifier based on Bayes' theorem. The algorithm first estimates the conditional probability of each variable from the training set, and finally finds the output with the largest posterior probability. The advantage of the GNB model is that it has a stable classification efficiency, a relatively simple algorithm, and performs well on small-scale data. |
| **MLP** | activation: relu hidden_layer_sizes: (10, 10) max_iter: 100 | Multi-layer perceptron (MLP) is a multi-layer feedforward neural network. The MLP model continuously improves the parameters in the hidden layer through the comparison and feedback of the output data with the actual situation, so as to achieve the highest prediction accuracy. |
| **KNN** | n_neighbors: 20 weights: uniform | KNN means that in the feature space, if most of the k nearest (ie nearest neighbors in the feature space) samples near a sample belong to a certain category, the sample also belongs to this category. |
| **SVM** | C: 1  kernel: rbf tol: 0.1 | The learning goal of SVM is to find a maximum interval plane in the projected high-dimensional space, so that the distance R from the support vector to the hyperplane is the largest. In order to deal with the explosive calculation problem in high-dimensional space, SVM introduces a kernel function to reduce the high-dimensional computation. SVM model has unique advantages in dealing with nonlinear separable, high-dimensional data classification problems and generalization ability. |
| **LR** | C: 1 max_iter: 100 penalty: l2 tol: 0.0001 | Logistic regression (LR) is a commonly used statistical classification model that uses a logistic function to model a binary dependent variable. Baseline factors performed significant difference between two groups were bring in model as adjusted variables. Use binary LR to evaluate the factors associated with lymph node metastasis, odds ratios (OR) and 95% confidence intervals (CIs) were determined in next. |
